# The Implication of 5-HT Receptor Family Members in Aggression, Depression and Suicide: Similarity and Difference

**DOI:** 10.3390/ijms23158814

**Published:** 2022-08-08

**Authors:** Nina K. Popova, Anton S. Tsybko, Vladimir S. Naumenko

**Affiliations:** The Federal Research Center Institute of Cytology and Genetics, Siberian Branch of the Russian Academy of Sciences, Novosibirsk 630090, Russia

**Keywords:** brain serotonin system, serotonin receptors, behavior, aggression, depression, suicide

## Abstract

Being different multifactorial forms of psychopathology, aggression, depression and suicidal behavior, which is considered to be violent aggression directed against the self, have principal neurobiological links: preclinical and clinical evidence associates depression, aggression and suicidal behavior with dysregulation in central serotonergic (5-HT) neurotransmission. The implication of different types of 5-HT receptors in the genetic and epigenetic mechanisms of aggression, depression and suicidality has been well recognized. In this review, we consider and compare the orchestra of 5-HT receptors involved in these severe psychopathologies. Specifically, it concentrates on the role of 5-HT_1A_, 5-HT_1B_, 5-HT_2A_, 5-HT_2B_, 5-HT_2C_, 5-HT_3_ and 5-HT_7_ receptors in the mechanisms underlying the predisposition to aggression, depression and suicidal behavior. The review provides converging lines of evidence that: (1) depression-related 5-HT receptors include those receptors with pro-depressive properties (5-HT_2A_, 5-HT_3_ and 5-HT_7_) as well as those providing an antidepressant effect (5-HT_1A_, 5-HT_1B_, 5-HT_2C_ subtypes). (2) Aggression-related 5-HT receptors are identical to depression-related 5-HT receptors with the exception of 5-HT_7_ receptors. Activation of 5-HT_1A_, 5-HT_1B_*,* 5-HT_2A_, 5-HT_2C_ receptors attenuate aggressiveness, whereas agonists of 5-HT_3_ intensify aggressive behavior.

## 1. Introduction

Aggression, depression and suicide are the global burden of human society. Each year, almost one million people die of suicide [1,2]. Major Depressive Disorder (MDD) is one of the most frequent psychiatric disorders affecting 5–12% of men and 10–25% of women [3]. Acts of violence and aggression account for 1.43 million deaths worldwide annually [4].

Despite being different multifaceted forms of psychopathology, aggression, depression and suicidal behavior nevertheless have some neurobiological links: (1) suicidal attempts are considered as violent aggression directed against the self [5], and associated with depression [6], violence and impulsive-aggressive behavior [7,8,9]. Analysis of literature revealed that 23 out of 37 variables that were considered as risk factors for violence are risk factors for suicide as well [10]. In murderers, the incidence of suicide is extraordinarily high, amounting to 30 percent in some European countries [11,12]. (2) Widely accumulated preclinical and clinical evidence associates depression [13,14,15], aggression [16,17,18] and suicidal behavior [1,9,19,20,21,22] with dysregulation in central serotonergic (5-HT) neurotransmission. Currently, the brain 5-HT system is the main target for antidepressant drugs: almost all clinically effective antidepressants act through 5-HTergic neurons (the only exception—bupropion) [23].

Polyfunctionality of the brain 5-HT is due to impressive variety of 5-HT receptors. Currently, 14 types of 5-HT receptors have been cloned and identified including both metabotropic G-protein-coupled and inotropic (5-HT_3_) receptors. Distinct types of 5-HT receptors are targets for approximately 40% of approved medicines [24]. Available 5-HT receptor density data suggest that the antidepressant effect of serotonin-selective reuptake inhibitors (SSRIs) is only observable when inhibitory and excitatory 5-HT receptors are balanced [25].

The crucial and intriguing problem is to define the similarity and the difference in the ensemble of 5-HT receptors regulating the specific and multifactorial kinds of psychopathology, such as aggression, depression and suicidal behavior. The aim of this review is to evaluate the contribution of the members of 5-HT receptor superfamily different by operational (drug-related), transductional (receptor coupling) and structural (primary amino acid sequence) characteristics (5-HT_1A_, 5-HT_1B_, 5-HT_2A_, 5-HT_2B_, 5-HT_2C_, 5-HT_3_ and 5-HT_7_) to the regulation of these kinds of behavior.

## 2. The 5-HT_1A_ Receptor

The 5-HT_1A_ receptor attracts special attention due to its key role in the autoregulation of the brain 5-HT system functional activity. The net effect of 5-HT_1A_ signaling is to reduce neuronal firing rate and protein kinase activation [26]. However, according to its localization, the 5-HT_1A_ receptor exerts different effects on the functional states of the 5-HT system. 5-HT_1A_ receptors are found on 5-HT cell bodies and dendrites, mainly in the midbrain raphe nucleus region (presynaptically located autoreceptors) and on terminal targets of 5-HT release (postsynaptic 5-HT_1A_ receptors). Presynaptic 5-HT_1A_ receptors inhibit neuronal spike activity in dorsal raphe nucleus and 5-HT release into the synaptic cleft [27,28,29]. Negative feedback control of functional activity of 5-HT neurons by presynaptic 5-HT_1A_ receptors was considered as a key mechanism in the autoregulation of the brain 5-HT system. Postsynaptic 5-HT_1A_ receptors mediate the action of serotonin on neurons and also could regulate 5-HT system functional activity via complex feedback neural networks [30,31]. Thus, the 5-HT_1A_ receptors are a powerful regulator of both pre- and postsynaptic 5-HT neurotransmission involved in mechanisms of sleep, stress response, appetite, sexual motivation, aggressive behavior, depression and anxiety.

### 2.1. The 5-HT_1A_ Receptor in Aggressive Behavior

An inhibitory effect of 5-HT_1A_ receptor agonists on aggressive and social behavior was shown in different animal models [16,32,33,34,35,36,37]. Considerable differences in 5-HT_1A_ receptors were found between rats selectively bred for high levels of aggressive reaction towards man or for its absence [16,38]. Genetically defined aggressiveness was shown to be associated with decreased expression of 5-HT_1A_ receptor mRNA in the midbrain, decreased 5-HT_1A_ receptor density in hypothalamus, frontal cortex and amygdala and decreased functional activity of 5-HT_1A_ receptors. Notably, the greatest difference between aggressive and tame rats was found in the structures of cortico-limbic circuitry (frontal cortex, amygdala, and hypothalamus) representing neuroanatomical substrates for the origins and expression of impulsive aggressive behavior [4,39,40,41,42]. The frontal cortex-amygdala network supports affective control [43] and regulates aggressive impulses originating in the amygdala [42,44,45]. These data suggested an important role of 5-HT_1A_ receptors in the suppression of impulsive aggressive behavior, and are consistent with the studies carried out in man. There was a significant negative correlation found between lifetime aggression and binding potential of 5-HT_1A_ receptors measured by positron emission tomography (PET) [46] and by the response to 5-HT_1A_ receptor agonist, ipsapirone [47].

Positive correlation of reduced 5-HT_1A_ receptor binding in the temporal cortex with aggressive behavior in Alzheimer disease was described by Lai and co-authors [48], who suggested that the 5-HT_1A_ receptor B_max_ represented the best predictor for aggression. 

Taken together, the evidence reviewed above suggests that 5-HT_1A_ receptors contribute to prefrontal cortex-limbic system circuits and may fulfill a significant role in the expression of impulsive aggression. Hereditary high or low aggressiveness may be defined, at least partly, by the expression and density of 5-HT_1A_ receptors in the prefrontal cortex and limbic system.

### 2.2. The 5-HT_1A_ Receptor and Depression

A lot of pharmacological evidence on the role of 5-HT_1A_ receptors in the mechanisms underlying depression and depressive-like behavior are available. Antidepressant-like effects similar to those evoked by classic antidepressants from the SSRI family have been produced by agonists at 5-HT_1A_ receptors [49,50,51,52]. Moreover, the involvement of the 5-HT_1A_ receptor in antidepressant action of SSRIs is well established. It is known that SSRI administration blocks 5-HT transporters thus increasing 5-HT levels in the synaptic cleft and inhibiting 5-HT exocytosis via presynaptic 5-HT_1A_ receptor-mediated negative feedback. Applied chronically, SSRIs lead to desensitization of presynaptic 5-HT_1A_ receptors that weakens the inhibitory effect of these receptors on the 5-HT system, thereby increasing its functional activity and ameliorating depression [26]. It was suggested that the enhancement of 5-HT_1A_ receptor-induced signaling intensifies antidepressant-like effects of 5-HT_1A_ receptor activation [53]. Reduction of 5-HT_1A_ autoreceptors in adults results in elevated release of 5-HT at target areas and enhanced SSRI-mediated behavioral improvement of depression [54,55,56,57]. The drugs combining SSRI action and postsynaptic 5-HT_1A_ receptor agonists [58] or presynaptic 5-HT_1A_ receptor antagonists [59,60] were shown to be effective for amelioration of depressive behavioral traits. 

Conditional knockout of the 5-HT_1A_ receptor gene transcription repressor Freud-1 in 5-HT neurons resulted in elevated 5-HT_1A_ autoreceptor protein and hypothermic response in mice. These changes were accompanied with reduced 5-HT levels and neuronal activity in the dorsal raphe. Such mutant mice demonstrated anxiety- and depressive-like behavior that was resistant to chronic antidepressant (fluoxetine) treatment [61]. The loss of function of presynaptic 5-HT_1A_ autoreceptors results in a disruption to the response to SSRIs [62,63]. At the same time, AAV-based Freud-1 knockdown in the hippocampus affecting postsynaptic 5-HT_1A_ receptors resulted in an antidepressant-like effect [64]. Another 5-HT_1A_ repressor of interest is Deaf1. It was demonstrated that a polymorphism in the human promoter that disrupts Deaf1 binding as well as the modeling of this mutation in mice are in good agreement and in both cases are associated with a depressive phenotype [65].

SiRNA targeting 5-HT_1A_ receptor mRNA covalently binded with the SSRI sertraline in order to concentrate it in serotonin axons decreased the 5-HT_1A_ autoreceptor expression without affecting the postsynaptic 5-HT_1A_ receptor expression in the hippocampus and prefrontal cortex. This led to marked antidepressant-like effects in the forced swim and tail suspension tests [55].

Further evidence for the involvement of 5-HT_1A_ receptors in depression came from postmortem studies showing increased levels of 5-HT_1A_ autoreceptors in human depression [66]. In brain areas with postsynaptic 5-HT_1A_ receptor localization, the hippocampus and frontal cortex, a decrease in 5-HT_1A_ receptor gene expression was observed in postmortem studies of major depression [67]. PET imaging studies of patients with bipolar depression and MDD demonstrated decreased 5-HT_1A_ receptor density in the dorsolateral prefrontal cortex [68,69,70]. Later, reduction of 5-HT_1A_ receptor density was shown in the mesiotemporal cortex of depressed patients. Smaller reductions were also reported in 5-HT_1A_ receptor binding in the hippocampus, raphe nuclei, insular, anterior cingulate cortex and occipital cortex of people with depression [71].

### 2.3. The 5-HT_1A_ Receptor and Suicide

In contrast to numerous animal models of depressive and, especially, aggressive behavior, no convincing animal model of suicide has been produced to date [72]. The data concerning the implication of 5-HT receptors in suicidal behavior were obtained using postmortem brain tissue studies, genetic association of 5-HT receptor polymorphism, and using PET in patients with a history of suicide attempts or suicidal intentions.

Higher 5-HT_1A_ binding potential of 5-HT_1A_ autoreceptors was shown in raphe nuclei of individuals with depression who attempted suicide [66,73] and in the raphe 5-HT_1A_ receptor density of individuals who committed suicide [74,75]. No changes in 5-HT_1A_ receptor density in the prefrontal cortex of suicide victims were revealed [74]. At the same time, reduced somatodendritic and postsynaptic 5-HT_1A_ receptor numbers or affinity [76] and a decrease in activity of cortical 5-HT_1A_ receptor downstream effectors [77] in suicide victims have been reported. However, the fact that the post-mortem samples are largely composed of suicide victims, many of whom suffered from chronic alcoholism, weakens this argument. Recently, the association of suicide in MDD patients with disruption of cortical 5-HT_1A_ receptor functioning [78] was demonstrated. 

## 3. The 5-HT_1B_ Receptor

The 5-HT_1B_ receptor is a G_i_-protein-coupled adenylate cyclase-inhibiting receptor displaying 43% amine acid sequence homology with the 5-HT_1A_ receptor [28,29,79]. However, 5-HT_1A_ and 5-HT_1B_ receptors have shown different cellular localization and regional brain distribution. The cellular localization of 5-HT_1B_ receptors is mainly presynaptic, with receptors located primarily on axon terminals. Depending on localization, a 5-HT_1B_ receptor may act as a autoreceptor, inhibiting 5-HT release; or as a heteroreceptor, located on non-serotonergic neurons and regulating the release of other transmitters [29,80,81,82]. A comparison of the roles of 5-HT_1A_ and 5-HT_1B_ receptors in the regulation of extracellular 5-HT in different brain regions suggested that the 5-HT_1A_ autoreceptor plays a larger role in the striatum innervated by the dorsal raphe nucleus, whereas the role of 5-HT_1B_ receptors is greater in the hippocampus and other brain regions innervated by the median raphe nuclei [83]. A full review of the 5-HT_1B_ receptor is beyond the scope of the present article but has been well covered in the comprehensive review of Tiger and co-authors [82].

### 3.1. The 5-HT_1B_ Receptor in Aggressive Behavior

Several lines of evidence indicate the essential role of the 5-HT_1B_ receptor in the modulation of aggressive behavior: (1) lacking 5-HT_1B_ receptor knockout mice demonstrate enhanced aggressive behavior and reduced anxiety [84,85]. (2) Alcohol-heightened aggression [86] and socially provoked aggressive behavior [87] are highly sensitive to the inhibitory effect of 5-HT_1B_ agonists. Microinjection of the 5-HT_1B_ agonist, CP-94,253, into the dorsal raphe reduced both aggressive and motor behaviors in mice with alcohol-escalated aggression. However, infusion of the 5-HT_1B_ agonist into the medial prefrontal cortex after alcohol drinking increased aggressive behavior [88,89]. (3) Repeatedly observed aggression increased aggressiveness in rats [90,91]. These changes in aggressive behavior were accompanied by decreased 5-HT_1B_ receptor density in the striatal brain regions and increased 5-HT_1B_ receptor density in the basolateral amygdala suggesting a modulatory role of 5-HT_1B_ receptors in the mechanism of learned aggression. (4) The SNP rs6296 in the 5-HT_1B_ gene was associated with childhood aggressive behavior but not with adulthood anger and hostility [18].

### 3.2. The 5-HT_1B_ Receptor in Depressive Behavior and Suicide 

A review of the literature, albeit fraught with inconsistent results, provides strong evidence in support of the involvement of 5-HT_1B_ receptors in the pathophysiology of depression and in the action of classical antidepressants, SSRIs. Behavioral antidepressant-like effects similar to those induced by SSRIs have been produced by agonists of 5-HT_1B_ receptors [3,52,81,92,93]. Furthermore, 5-HT_1B_ receptor knockout or pharmacological blockade of 5-HT_1B_ receptors abolished the antidepressant effect of SSRIs [92], suggesting significant contribution of the 5-HT_1B_ receptors in the mechanisms of SSRIs action.

Overexpression of encoding 5-HT_1B_ receptor gene in the caudal dorsal raphe nucleus increased swimming in the swimming forced test and reduced conditioned freezing [94]. At the same time, decreased anxiety along with an antidepressant-like effect in the forced swim and sucrose preference test were displayed by mice lacking 5-HT_1B_ autoreceptors [95]. The antidepressant-like effect was produced by the 5-HT_1B_ receptor agonist anpirtoline [92]. Reduced 5-HT_1B_ receptor binding in ventral striatal/ventral pallidal brain regions has been reported in MDD [82,96]. Importantly, the firing of 5-HT neurons in the dorsal raphe nucleus is controlled by both 5-HT_1A_ and 5-HT_1B_ receptors. However, in contrast to the inhibitory influence of 5-HT_1A_ receptors, excitatory control of the 5-HT neurons firing through 5-HT_1B_ autoreceptors was revealed [97].

Attempts to find evidence for the implication of 5-HT_1B_ receptor genetic polymorphism in the susceptibility to suicide were unsuccessful [98,99,100,101,102,103], suggesting that 5-HT_1B_ polymorphism is unlikely to play a major role in the genetic predisposition to suicide attempts. However, in one more recent study, an association was shown between a few 5-HT_1B_ polymorphisms, MDD, suicide and aggression [104].

## 4. The 5-HT_2_ Receptor Family

Serotonin 2A (5-HT_2A_), 5-HT_2B_ and 5-HT_2C_ receptors are members of the superfamily of 7-transmembrane-spanning (7-TMS) receptors. These receptors share about 46–50% overall sequence identity and couple preferentially to G_q/11_ to increase inositol phosphates and cytosolic Ca^2+^ [105]. The 5-HT_2A_ receptors are predominantly cortical, and in subcortical structures their expression is considerably lower [106,107]. Cortical 5-HT_2A_ signaling can initiate a negative feedback mechanism through cortical glutamatergic and GABAergic interneurons that inhibits the firing of 5-HT neurons in the dorsal raphe nuclei [31]. At the membrane level, activation of 5-HT_2A_ receptors produced membrane depolarization and the closing of potassium channels that increased the excitability of host neuron [108].

One remarkable characteristic of 5-HT_2A_ and 5-HT_2C_ receptors is constitutive activity [109] revealed by the presence of receptor signaling in the absence of any ligand [110]. Constitutive activity of 5-HT_2A_ and 5-HT_2C_ receptors can impact and significantly change the therapeutic response of these receptors [109].

Widely presented in astrocytes, 5-HT_2B_ receptors play a key role in astrocyte response to antidepressant treatment. Upon stimulation, 5-HT_2B_ receptors activate MEPK/EKT and PI3K/AKT signal pathways via EGF receptor transactivation that leads to changes in the expression of multiple genes and affects astrocytic functions including, possibly, gliotransmitter secretion [111]. More intriguingly still, 5-HT_2B_ receptors are expressed in the 5-HT neurons and, acting as somatodendritic autoreceptors, regulate their excitability together with 5-HT_1A_ receptors [112]. Stimulation of the 5-HT_2B_ receptor is associated with an increase in cyclic GMP through the dual activation of constitutive and inducible Nitric Oxide Synthase [113,114]. 

Initially erroneously identified as 5-HT_1C_ receptors [115], 5-HT_2C_ receptors are found widely distributed throughout the brain [105]. The primary transcript of the 5-HT_2C_ receptor is subjected to multiple RNA editing. Fully edited variants (VSV and VGV) of 5-HT_2C_ receptors have reduced G-protein coupling and 40-fold decreased serotonergic potency [116]. Within the brain, 5-HT_2C_ receptors modulate the mesolimbic dopaminergic function exerting a tonic inhibitory influence over dopamine neurotransmission [117,118]. High levels of 5-HT_2C_ receptors were detected on parvalbumin GABAergic neurons in the prelimbic prefrontal cortex and to a lesser degree on pyramidal glutamatergic neurons [117].

### 4.1. The 5-HT2A Receptor

#### 4.1.1. The 5-HT_2A_ Receptor in Aggressive Behavior

There are some pharmacological data indicating a link between aggressive behavior and 5-HT_2A_ receptor activity. In animals, 5-HT_2A_ agonists, such as DOI, reduced aggressive behavior in flies, amphibians, mice and rats [34]. However, accumulated data also revealed a pro-aggressive effect of the 5-HT_2A_ agonist DOI [119,120], whereas 5-HT_2A_ antagonists effectively suppressed aggressive behavior [119,121,122]. 

In humans, a number of atypical antipsychotics, which act as antagonists of 5-HT_2A_ receptors, had antiaggressive effects in clinical trials reviewed by Comai and co-authors [123]. A number of polymorphisms associated with impulsivity, aggression and violence were reported [124,125,126].

Some conflicting results were obtained in PET studies. Compared with the low-IA (impulsive aggression) group, cortical 5-HT_2A_ receptors in the high-IA group were modestly lowered [127]. No differences between cortical 5-HT_2A_ receptor levels in high- and low-aggressive participants was found [128].

5-HT_2A_ receptor binding was increased in the hippocampus [129] and diminished in cortical areas and basal ganglia [130] of subjects with borderline personality disorder (BPD) characterized by impulsive aggression. In contrast, Rosell and co-authors [131] demonstrated positive association of cortical 5-HT_2A_ receptor binding in physically aggressive BPD subjects. Positive correlation of prefrontal 5-HT_2A_ receptor binding with lifetime history of aggression was found in a postmortem study of suicide victims [132].

#### 4.1.2. The 5-HT_2A_ Receptor in Depressive Behavior and Suicide

The 5-HT_2A_ receptor is the primary site of the action of 5-HTergic hallucinogens, such as LSD, psilocybin, mescaline, currently recognized as fast acting antidepressants [133]. Depressive-like behavior was not affected in mice with global knockout of 5-HT_2A_ receptor [134]. However, in response to chronic corticosterone exposure, *Htr2a*^−/−^ mice displayed a more pronounced anxiodepressive-like phenotype than wild-type mice [135]. 

Selective 5-HT_2A_ antagonists generate antidepressant-like effects, inhibiting 5-HT reuptake and modulating the release of other neurotransmitters in the prefrontal cortex [52,136,137]. Numerous open-label and placebo-controlled studies have suggested that some antidepressants and atypical antipsychotic drugs known to block 5-HT_2A_ receptors augment the clinical response to SSRIs in treatment-resistant patients [59,136]. 

Committed suicide depressive patients show increased expression of 5-HT_2A_ receptors in the prefrontal cortex and both lower expression and reduced 5-HT_2A_ receptor binding affinity in the hippocampus compared with matched controls [138]. In fact, results of studies on 5-HT_2A_ binding have been equivocal depending on the character of suicide, brain region and diagnosis, as reviewed by Stockmeier [139]. Deliberate self-harm patients had a significantly reduced 5-HT_2A_ frontal binding index. The reduction was more pronounced among self-injury patient than among self-poisoning patients [140]. In the recent study by Underwood and co-authors [141], the 5-HT_2A_ binding was greater in the prefrontal cortex of MDD suicides with alcoholism and childhood adversity. Evidence from direct in vivo functional imaging with either PET or Single-Photon Emission Computed Tomography demonstrated contradicting results with lower [142,143,144], unchanged [145,146] and higher [147] levels of 5-HT_2A_ binding in MDD patients.

Despite the huge amount of studies, the contribution of 5-HT_2A_ polymorphisms to depressive disorders in humans is not fully understood. The number of meta-analyses did not show any significant association between polymorphisms in the *Htr2a* gene and depressive disorders [148,149,150]. However, recent gene-based analysis does suggest an association of the *Htr2a* gene with antidepressant treatment response in depressed patients [151,152].

The majority of studies devoted to finding a link between 5-HT_2A_ receptor polymorphisms and suicidal risk failed to find any association [153,154,155,156,157]. At the same time, in a number of studies, an association between the *Htr2a* gene variants and suicidal behavior in subjects with stressful life events [158,159], such as sexual and physical child abuse, was found [160]. 

### 4.2. The 5-HT_2B_ Receptor in Aggressive and Depressive Behavior

It has been generally assumed that 5-HT_2B_ receptor dysfunction or deficiency resulted in increased impulsivity and aggression. High impulsivity was found in 5-HT_2B_ mutant (*Htr2b*^−/−^) mice [161]. Among the QTLs underlying behaviors associated with intermale aggression in mice, the strongest candidate within the narrow QTL interval on chromosome 1 for both attack and latency variables is *Htr2b* gene [162].

Humans with specific 5-HT_2B_ receptor stop codon (*Htr2b* Q20*), that led to loss of receptor expression, are predisposed to severe impulsivity and aggressive behavior towards themselves and others [163,164,165]. Genomic-wide association studies and experiments on 5-HT knockout mice implicate the 5-HT_2B_ receptor as a major locus associated with cannabis-induced aggression both in mice and humans [166].

**Figure 1:**
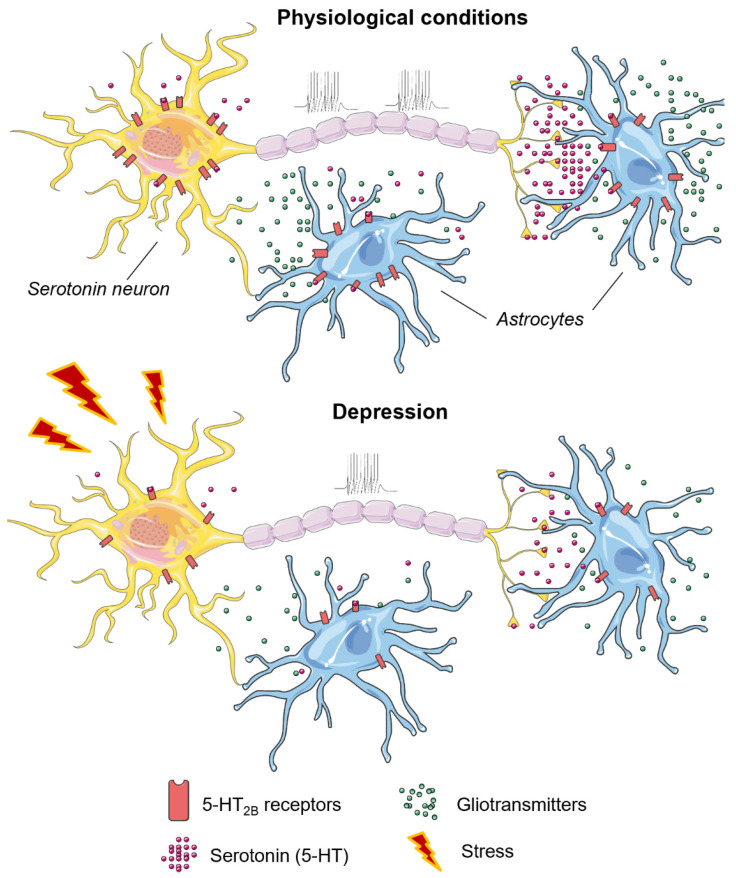
Hypothetical mechanism of 5-HT_2B_ receptors implicated in the mechanisms of depression. Under physiological conditions, 5-HT_2B_ receptors directly modulate serotonergic neurotransmission as well as astrocytic functions; under stressful conditions, the 5-HT_2B_ receptors are downregulated which may lead to both serotonergic and astrocytic dysfunctions.

A lack of SSRI effects was observed in *Htr2b*^−/−^ [167] and *Htr2b*^5-HTKO^ mice [112,168]. In contrast, agonist-induced stimulation of 5-HT_2B_ receptors mimicked behavioral and neurogenic SSRI actions [167]. Of interest was that non-stressed 5-HT_2B_ knockout mice displayed an antidepressant-like phenotype that was reversed to depressive-like after four weeks of social isolation [169]. There is much evidence that astroglial, rather than neuronal 5-HT_2B_ receptor expression changes are associated with depressive behaviors [111]. Recently it was found that down-regulation of astrocytic 5-HT_2B_ receptors may underlie depressive-like behavior induced by sleep deprivation, while restoration of receptor levels augments the antidepressant action [170]. Based on the existent literature data, we suggested a hypothetical mechanism of 5-HT_2B_ receptors implicated in the mechanisms of depression (Figure 1).

### 4.3. The 5-HT2C Receptor

#### 4.3.1. The 5-HT_2C_ Receptor in Aggressive Behavior

The role of 5-HT_2C_ receptors in aggressive behavior has long remained elusive due to the lack of selective ligands [9]. To our knowledge, the first evidence of the implication of 5-HT_2C_ receptors in aggressive behavior was obtained in our experiments on rats selected for many generations for high or low impulsive aggressiveness [171]. Significant difference between highly aggressive and nonaggressive rats in the expression and functional response of 5-HT_2C_ receptors was shown. The level of 5-HT_2C_ receptor mRNA in the frontal cortex and hippocampus and functional response to 5-HT_2C_ receptor agonist was lower in aggressive rats than in nonaggressive animals, suggesting an inhibitory role of 5-HT_2C_ receptors in genetically-defined aggressiveness. 

There are a few currently available data in support of the antiaggressive role of 5-HT_2C_ receptors: (1) the activation of 5-HT_2C_ receptors enhanced the display of defeat submissive and defensive behavior in golden hamsters [172]. (2) 5-HT_2C_ receptor agonist/alpha 2 receptor antagonist S32212 suppressed aggressive behavior in mice [173]. (3) Mice expressing only the VGV isoform of 5-HT_2C_ receptors displayed a high level of conspecific aggression [174]. (4) The association between *Htr2c* gene polymorphism and criminal behavior in humans was demonstrated [175]. (5) Recently, a novel 5-HT_2C_ agonist, lorcaserin, has been demonstrated to have antiaggressive properties in human subjects with impulsive aggressive behavior. Lorcaserin attenuated provoked, but not unprovoked, aggression in impulsively aggressive individuals indicating that 5-HT_2C_ receptor may be a putative target for the treatment of impulsive aggressive behavior in human subjects [176].

#### 4.3.2. 5-HT_2C_ Receptor, Depressive Behavior and Suicide

*Htr2c*^−/−^ mice do not exhibit depressive-like behavior in a TST paradigm [177]. However, 5-HT_2C_ knockout enhanced fluoxetine effects on immobility in the TST [177]. Recently, Demireva and co-authors demonstrated that 5-HT_2C_ receptor blockade led to augmentation of therapeutic antidepressant and anxiolytic effects of SSRIs [178]. Indeed, tricyclic antidepressants and SSRIs act as antagonists of 5-HT_2C_ receptors, and when administered chronically, can lead to 5-HT_2C_ receptor downregulation [179,180,181,182,183]. 5-HT_2C_ receptor antagonists not only possess antidepressant and anxiolytic properties in diverse rodent models [184], but are also introduced as antidepressant drugs in clinics. One of them, agomelatine, has long been registered for the treatment of MDD [185]. On the other hand, 5-HT_2C_ agonists also have antidepressant activity in various models of depressive-like behavior [186,187,188]. At least one of the explanations of paradoxical antidepressant-like effects of both agonists and antagonists of 5-HT_2C_, as well as 5-HT_2A_ receptors, is an impact of the constitutive activity of these receptors [109,110]. Constitutive activity of 5-HT_2A_ and 5-HT_2C_ receptors is identified by receptor signaling in the absence of any ligand and it can change the response to drugs. 

The role of 5-HT_2C_ gene polymorphism in mood disorders has also been investigated. In many studies, an association between Ser23 allele of rs6318 SNP, MDD and BD as well as antidepressant response was found [189,190,191,192,193]. Postmortem analyses of 5-HT_2C_ receptor mRNA-editing profiles in the whole brain and hypothalamus [194], the prefrontal cortex [195,196,197,198,199,200] and the anterior cingulate cortex [201] in suicides with psychiatric disorders like MDD, schizophrenia and bipolar disorder consistently showed increased levels of these epigenetic modifications regardless of the underlying disease. Nevertheless, an association between *Htr2c* gene variants and suicidal behavior was not confirmed in the majority of studies [202,203,204,205,206].

**Figure 2:**
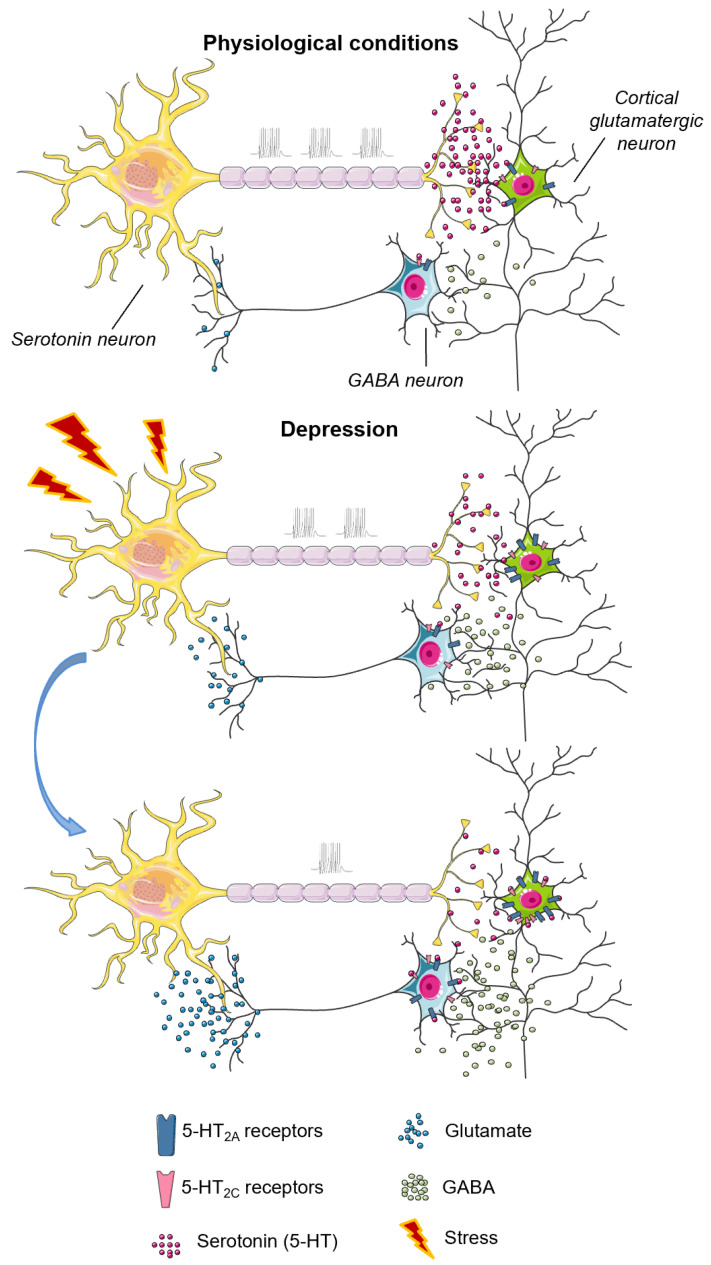
Schematic representation of mechanisms of 5-HT_2A_ and 5-HT_2C_ receptor involvement in the pathogenesis of depression. Under physiological conditions, postsynaptic 5-HT_2A_ and 5-HT_2C_ receptors regulate glutamate and/or GABA release; upon stress-induced serotonin depletion, 5-HT_2A_ and 5-HT_2C_ receptors are upregulated and sensitized. Sensitized 5-HT_2A_ and 5-HT_2C_ receptors indirectly inhibit serotonergic neurotransmission, aggravate serotonin deficit and provoke depressive-like behavior.

The sum of data indicates the opposite roles of 5-HT_2A/2C_ and 5-HT_2B_ receptors in the regulation of affective behavior. The 5-HT_2B_ receptors play an inhibitory role in both aggressive and depressive-like behavior, acting through direct modulation of serotonergic neurotransmission as well as astrocytic functions. Under stressful conditions, the 5-HT_2B_ receptors are downregulated, in contrast to 5-HT_2A_ and 5-HT_2C_ receptors that are upregulated and sensitized in response to stress. In turn, sensitized 5-HT_2A_ and 5-HT_2C_ receptors indirectly inhibit serotonergic neurotransmission and provoke depressive-like behavior (Figure 2). At the same time, 5-HT_2A/2C_ receptors play an opposite role in the regulation of impulsivity.

## 5. The 5-HT_3_ Receptor

The 5-HT_3_ receptor is the only known exception among G-protein-coupled receptors in the 5-HT receptor family. Unlike all the others 5-HT receptors, the 5-HT_3_ receptor is a ligand-gated ion channel. It belongs to the Cys-loop receptor family of pentametric neurotransmitter-gated ion channels permeable to Ca^2+^, Na^+^ and K^+^, and plays a key role in fast synaptic transmission. The 5-HT_3_ receptor expressing neurons are mainly GABA cells in the neocortex, olfactory cortex, hippocampus and amygdala [207]. It was suggested that the activation of 5-HT_3_ receptors inhibits pyramidal neurons in the medial prefrontal cortex via GABAergic interneurons [208]. In addition, 5-HT_3_ receptors control dopamine and acetylcholine release, and this interrelation can be an important mechanism the 5-HT_3_ receptor ligands effects [60].

The most well established physiological roles of the 5-HT_3_ receptor are to regulate gastrointestinal motility and coordinate emesis and vomiting [60,209]. Thus, 5-HT_3_ agonists cause unpleasant effects of nausea, vomiting and anxiety, and have not been used clinically owing to their emetogenic and anxiogenic properties [210]. Additionally, it was shown that central 5-HT_3_ receptors play an important role in thermoregulation [211,212].

Meanwhile, 5-HT_3_ antagonists produced distinct antiemetic activity for chemotherapy-induced vomiting and different kinds of chronic neuropathic nausea and vomiting [213]. Antagonists of 5-HT_3_ receptors do not modify any aspects of normal behavior in animals or induce pronounced changes in physiological functions in healthy subjects [213]. The efficacy was shown mainly in pathological models of behavior [214]. Positive anti-inflammatory and immunomodulatory effects of 5-HT_3_ antagonists (seemingly related to substance P-mediated inflammation and hyperalgesia) have also been observed [210].

### 5.1. The 5-HT_3_ Receptor in Aggression

Antagonists of 5-HT_3_ receptors—usually referred to as setrons (ondansetron, zacopride, tropisetron)—reduced alcohol-heightened aggression in mice [215], apomorphine-induced aggressive behavior in rats [216], and aggressive response of cocaine-treated hamsters, whereas 5-HT_3_ receptor agonist mCPBG stimulated aggressive behavior in hamsters [217].

The 5-HT_3_ receptor density was greater in highly aggressive (H-Agg) compared with low-aggressive (L-Agg) hamsters [218]. No significant effect of 5-HT_3_ overexpression on aggressive behavior was found. However, ondansetron and zacopride reduced intermale aggression in both B6SJL/F2 transgenic 5-HT_3_ overexpressing and wild-type mice [215].

These data showed the implication of the 5-HT_3_ receptor in the regulation of aggressiveness and suggested the 5-HT_3_ receptor as a pro-aggressive factor [218,219]. However, this suggestion met some controversies: (1) isolation-induced aggressive behavior is accompanied by down-regulated hypothalamic 5-HT_3_ protein level. (2) Intrahypothalamic infusion of ondansetron increased isolation-induced aggression, whereas 5-HT_3_ receptor agonist SR57227A decreased aggression levels [220]; and 5-HT_3_ antagonist zacopride failed to attenuate isolation-induced aggression [122]. It therefore seems that the antiaggressive effect of the 5-HT_3_ receptor antagonists is dependent upon the phenotype. Tropisetron inhibited expression of aggression in an impulsive-aggressive phenotype High-Aggression group of golden hamsters, while enhancing aggressive behavior in Low-Aggressive animals [219]. The aggression-reducing effect of 5-HT_3_ receptor antagonists was found in the offensive response of adolescent cocaine-treated hamsters [217] and alcohol heightened aggression [215]. Taken together these data show 5-HT_3_ receptor antagonists to be a promising antiaggression substance, although this effect depends on the genetic background of the animal and on the kind of aggression in question. 

### 5.2. The 5-HT_3_ Receptor in Depression

Accumulated evidence that is well covered in comprehensive reviews [60,214,221,222] suggested 5-HT_3_ receptor antagonists as possible antidepressant drug targets. Indeed, 5-HT_3_ receptor antagonists inhibit the binding of 5-HT to postsynaptic 5-HT_3_ receptors and increase their availability to other receptors like 5-HT_1A_, 5-HT_1B_ and 5-HT_2A_ receptors, thereby producing an antidepressant effect [222]. Antidepressant-like effects of 5-HT_3_ receptor antagonists ondansetron, zacopride, ICS 205-930 [181,223,224] and tropisetron [225] were demonstrated on mice and rats in various behavioral models of depression. 

### 5.3. The 5-HT_3_ Receptor in Suicidal Behavior

In contrast to numerous data demonstrating the link between the 5-HT_3_ receptor, aggression and depression, investigations in to the involvement of the 5-HT_3_ receptor in suicidal behavior are scarce. The few currently available studies give a reason to believe that 5-HT_3_ receptors are not involved in the predisposition to suicide. In particular, no differences in number and affinity of 5-HT_3_ receptors in the cortex of suicide victims were shown [226]. The data concerning 5-HT_3A_ and 5-HT_3B_ receptor polymorphisms also suggest that 5-HT_3_ receptors may not play a major role in the susceptibility to suicidal behavior in schizophrenia patients [227].

## 6. The 5-HT_7_ Receptor

The 5-HT_7_ receptor is one of the most recently described G-protein-coupled 5-HT receptors. This receptor exhibits a high percentage of homology with the 5-HT_1A_ receptor and exerts its effects on neurons via the same second messenger as the 5-HT_1A_ receptor—adenylyl cyclase. However, the 5-HT_7_ receptor activates adenylyl cyclase, whereas the 5-HT_1A_ receptor inhibits it.

### 6.1. The 5-HT_7_ Receptor in Aggression

In contrast with very consistent lines of evidence that the 5-HT_7_ receptor contributes to modulatory mechanisms of depression, efforts to evaluate the implication of the 5-HT_7_ receptor in the control of aggressive behavior have been negative. To our knowledge, there are no studies establishing a link between the 5-HT_7_ receptor and aggression. Administration of different doses of selective 5-HT_7_ receptor antagonist SB269970 to mice did not produce any significant effect on isolation-induced aggressive behavior [228]. In our experiments (unpublished data), no effect on intermale aggression in mice was found of intracerebroventricularly administered 5-HT_7_ receptor agonist, LP 44. 

### 6.2. The 5-HT_7_ Receptor in Depression

Studies utilizing 5-HT_7_ antagonists demonstrated the involvement of 5-HT_7_ receptors in the control of learning, circadian rhythmicity, sleep-disorders, mood and thermoregulation [229,230,231,232]. 

Converging lines of evidence suggested that 5-HT_7_ receptors contribute to genetic and physiological control of depressive behavior: (1) various 5-HT_7_ receptor antagonists including lurasidone [229,233], SB-269970 [234,235,236], SB-258719 [234], and JNJ-18038683 [237] produced antidepressant-like activity in the tail suspension and in the forced swimming tests; (2) 5-HT_7_ receptor antagonism has been posited as necessary for antidepressant activity of antipsychotic amisulpride [238,239]; (3) 5-HT_7_ knockout mice also showed decreased immobility in both Porsolt’s and tail suspension tests [234,235]; (4) chronic antidepressant treatment leads to decreased 5-HT_7_ receptor binding [240].

Remarkable coincidence of the effects of antidepressant treatment, 5-HT_7_ knockout and pharmacological blockade of 5-HT_7_ receptors indicates that 5-HT_7_ receptor facilitates the mechanisms provoking depression and suggest that 5-HT_7_ antagonists might have therapeutic value as novel antidepressant drugs [235,241,242]. Moreover, a novel antipsychotic drug lurasidone which is notable for high affinity for 5-HT_7_ receptor is approved for the treatment of schizophrenia and patients with major depressive episodes associated with bipolar depression in a number of countries including UK, USA, Canada and Australia [243].

Recently, a novel role of 5-HT_7_ receptors in the functionality of the 5-HT system was revealed. The idea that G-protein-coupled receptors (GPCRs) can function as dimers is now generally accepted [244,245,246]. Moreover, a growing body of evidence points to the functional importance of oligomers for receptor trafficking, receptor activation and G-protein coupling in native tissues [246]. The clinical significance of GPCR oligomerization has also become more evident during recent years, leading to identification of oligomeric complexes as novel therapeutic targets [247,248].

Convincing evidence indicating that G-protein-coupled 5-HT receptors can interact with each other forming protein-protein complexes has been obtained. It was found that 5-HT_1A_ receptors form heterodimers with 5-HT_7_ receptors (5-HT_1A_-5-HT_7_) [249,250]. Functionally, heterodimerization inhibits the binding of 5-HT_1A_ receptors to the G_i_-protein, reducing the 5-HT_1A_ receptor-mediated potassium channel activation, and facilitates the internalization of 5-HT_1A_ receptors without affecting the 5-HT_7_ receptor-mediated signaling [250]. Thus, the formation of the 5-HT_1A_-5-HT_7_ receptor complex enhances the desensitization of 5-HT_1A_ receptors with unchanged 5-HT_7_ receptor functional activity. Although the evidence for the physiological significance of 5-HT_1A_-5-HT_7_ dimers has been obtained mainly from cell culture experiments, taking into account the acknowledged role of 5-HT_1A_ receptors in autoregulation of the brain 5-HT system, these data suggest 5-HT_7_ receptors to be important regulators of 5-HT_1A_ activity.

**Figure 3:**
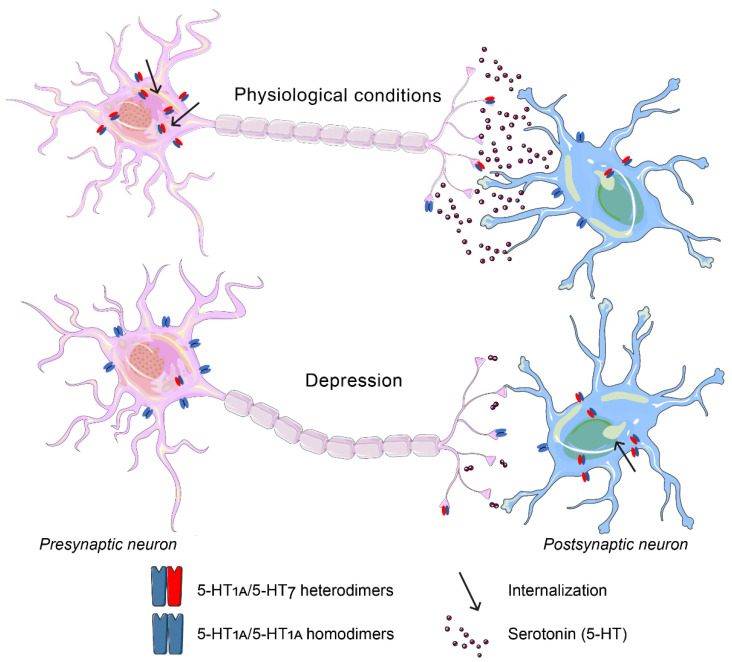
Hypothetical mechanism of the role of 5-HT_1A_/5-HT_7_ receptor heterodimerization in the mechanism of depression. Under physiological conditions, the amount of 5-HT_1A_/5-HT_7_ heterodimers in presynaptic neurons is higher than in postsynaptic neurons; under depression, the 5-HT_1A_/5-HT_1A_ homo- and 5-HT_1A_/5-HT_7_ heterodimers ratio in presynaptic neurons shifts towards 5-HT_1A_/5-HT_1A_ homodimers which decreases the amount of 5-HT in the synaptic cleft.

The possible role of 5-HT_1A_/5-HT_7_ heterodimers in the development of pathophysiological processes in the central nervous system and in the effect of antidepressant treatment is of particular interest. We suggested that the higher sensitivity of presynaptic 5-HT_1A_ receptors to prolonged 5-HTstimulation compared to postsynaptic 5-HT_1A_ receptors is based on the larger density of 5-HT_1A_/5-HT_7_ heterodimers on the presynaptic membrane [251]. According to this hypothesis, the ratio of 5-HT_1A_/5-HT_1A_ homodimers and 5-HT_1A_/5-HT_7_ heterodimers on pre- and postsynaptic terminals is not the same: 5-HT_1A_/5-HT_7_ heterodimeric complexes predominate on presynaptic terminals (Figure 3). SSRIs increase the level of 5-HT in the synaptic cleft, which enhances the internalization of 5-HT_1A_/5-HT_7_ receptor complexes. This leads to inhibition of the 5-HT_1A_ autoreceptor activity resulting in the increase in 5-HT system functional activity. Therefore, the formation of the 5-HT_1A_/5-HT_7_ heterodimeric complex may play a significant role both in the development of depression and in the mechanism of its treatment. We also suggested that under depression, the ratio of 5-HT_1A_/5-HT_1A_ homo- to 5-HT_1A_/5-HT_7_ heterodimers in presynaptic neurons may shift towards 5-HT_1A_/5-HT_1A_ homodimers, leading to a delay in 5-HT_1A_ autoreceptor internalization following SSRI treatment which could result in antidepressant resistance. If this hypothesis is correct, then artificial increase in 5-HT_7_ receptor expression in the raphe nuclei area should lead to a shift in the ratios of 5-HT_1A_/5-HT_1A_ homodimers and 5-HT_1A_/5-HT_7_ heterodimers towards 5-HT_1A_/5-HT_7_ heterodimers, enhance 5-HT_1A_ autoreceptor internalization and thus result in an antidepressant effect. Recently we verified this hypothesis, and showed that 5-HT_7_ receptor overexpression in the raphe nuclei area of the midbrain of both “nondepressive” C57Bl/6J mice and ASC mice with genetic predisposition to depressive-like behavior produced an antidepressant effect [252]. 

Meanwhile, despite comprehensive evidence on the implication of the 5-HT_7_ receptor in mood disturbances and MDD, there is no data on its role in the mechanisms underlying suicidal behavior. 

## 7. Discussion

Deepening acquaintance with functional characteristics and the nature of the participation of individual 5-HT receptors in the regulation of pathological behavior is important not only for our understanding of the mechanisms regulating pathological aggressiveness, depression, and suicide, but also for creating new, more effective antidepressant and antiaggressive pharmacological medicines. Over the past decade, a number of attempts have been made to enhance antidepressant effects by combining drugs that increase serotonergic activity. The combined drug approach is based on the utilization of different pathways enhancing 5-HT signaling—increased 5-HT synthesis, inhibited 5-HT reuptake and catabolism, and increased receptor activity—as well as on the implementation of specific receptor agonists, or blockade with antagonists.

A successful example of this novel approach is the unique multimodal 5-HTergic drug vortioxetine, which combines a 5-HT reuptake inhibitor activity with agonistic 5-HT_1A_ receptor activity and antagonistic 5-HT_3_ and 5-HT_7_ receptor activity. Preclinical and clinical trials have shown high efficacy of vortioxetine in MDD [253,254,255]. Vortioxetine was the first antidepressant to demonstrate clinical efficacy in improving cognition regardless of the effect on affective symptomatology [256,257]. Another advantage of vortioxetine over currently utilized antidepressants is a favorable effect in elderly patients [258]. Despite a large number of studies supporting vortioxetine, its place among antidepressants remains unclear due to the insufficient number of studies devoted to comparison with currently used antidepressants, primarily with the drugs from the SSRI group [259].

The similar effect of SSRIs and some 5-HT receptors on depressive and aggressive behavior, i.e., suppressive effect of 5-HT_1A_ receptors and facilitative 5-HT_3_ receptors (in absence of a significant effect of the 5-HT_7_ receptor on aggressiveness), suggests that vortioxetine should also have antiaggressive agent properties. Indeed, preliminary results support this assumption although so far obtained only for a very small number of patients [260].

Two main limitations of the multimodal drug approach are (1) potential danger of unwanted side-effects caused by increased action of a particular drug, e.g., hallucinogenic effects of 5-HT_2A_ receptor activation, emesis and vomiting produced by activation of 5-HT_3_ receptors; (2) the danger of the Serotonin Syndrome (SS) development—toxic symptoms produced from too much 5-HT in the central and peripheral nervous system [261,262].

Nevertheless, the key role of 5-HT in the regulation of behavior and mechanisms underlying a wide range of neuro- and psychopathologies, on the one hand, and the polyfunctionality and diversity of 5-HT receptors on the other hand, open broad prospects for the creation of new effective combined 5-HTergic drugs. As an example, an antidepressant drug litoxetine was developed, combining SERT inhibition and 5-HT_3_ antagonism to prevent SSRI-induced gastrointestinal side effects [263]. However, our growing knowledge of the role of 5-HT also highlights the necessity for a detailed investigation into the functional characteristics of all 5-HT receptor types as well as their cross-talk and roles in the regulation of numerous types of behavior.

## 8. Conclusions

Aggression, depression, and suicide are multifactorial behavioral conditions controlled by an ensemble of 5-HT receptor types exerting reciprocal suppressive or facilitative influence. 

Currently, the brain 5-HT system is the main target for antidepressant drugs: almost all clinically effective antidepressants act via 5-HTergic neurons (the only exception being bupropion) [23]. Available 5-HT receptor density data suggest that the antidepressant effect of SSRIs is only observable when inhibitory and excitatory 5-HT receptors are balanced [25]. 

This review examines the evidence for the contribution of seven types of 5-HT receptors to the regulation of aggressive, depressive and suicidal behavior in an attempt to identify similarities and differences in the 5-HT receptor ensemble underlying these psychopathologies. 

Comparison of aggressive and depressive behavior reveals significant similarities in the 5-HT receptors set and in the character of their modulating effect suggesting that impulsive violent aggressive behavior and depression share common genetic and epigenetic mechanisms. Along with 5-HT_1A_, 5-HT_1B_, and 5-HT_2B_ receptors, the activation of which produces antidepressant effects and suppresses (decreases) aggressiveness, 5-HT_3_ receptor agonists enhance both aggressiveness and depressive-like behavior (Table 1). The differences might be found in the effect of 5-HT_7_ receptor agonists, which enhance depression and, apparently, do not play a significant role in the regulation of aggressive behavior.

Paradoxically, an antidepressant effect may be produced by both agonists and antagonists of 5-HT_1B_, 5-HT_2A_ and 5-HT_2C_ receptors (Table 1), suggesting posttranslational modification due to the regional differences. Additionally, for these receptor subtypes, biased agonism was demonstrated. This phenomenon results in activation of different signal pathways depending on ligand [24,264,265,266]. At the same time, for 5-HT_2C_ receptors, mRNA-editing resulting in the translation of various isoforms of receptor was shown [267,268,269]. These features could also explain the paradoxically antidepressant effect induced by both agonist and antagonist of these receptors. Moreover, this rather disappointing situation of combined effects may be associated with the constitutive features of these types of 5-HT receptors, which are active even in the absence of a ligand. This may determine the unusual response of 5-HT_2A_/_2C_ receptors to antagonists [270].

The investigation of the individual roles of different 5-HT receptors in the mechanisms underlying suicide is complicated—not only by the great diversity of suicidal behavior (suicide ideation, suicidal attempt, completed suicide, depressive or violent suicide), but also, most importantly, by the lack of experimental animal model of suicide [72]. Nevertheless, the available data indicate the involvement of at least some of the 5-HT receptors in the mechanisms of suicide. This applies in particular to the main autoregulator of the brain 5-HT system—the 5-HT_1A_ receptor. An increase in the 5-HT_1A_ autoreceptor density in the raphe nuclei area of individuals who attempted suicide [66,73] and who committed suicide [74] was found. Indeed, 5-HT_1A_ receptors in the in raphe nuclei act as somatodendritic autoreceptors, and an increase in their activity leads to a decrease in 5-HT signaling in the brain that is in good agreement with the prevailing ideas about the role of 5-HT deficiency in psychopathologies. No changes in 5-HT_1A_ receptor density in the prefrontal cortex of suicide victims were revealed [74]. However, a decrease in activity of cortical 5-HT_1A_ receptor downstream effectors in suicide victims was shown [77]. Recently, the association of suicide with the disruption of cortical 5-HT_1A_ receptor functioning in MDD patients was demonstrated [78]. Another 5-HT receptor likely to be involved in mechanisms underlying suicide is the 5-HT_2A_ receptor. The 5-HT_2A_ receptor level in the frontal cortex is reported to be increased in suicide victims [21,141] but the 5-HT_2A_ receptor binding index in the frontal cortex of deliberate self-harm patients was decreased. An explanation of this discrepancy can be found in the study of Andenaert with co-authors [142], who showed that the nature of changes in 5-HT_2A_ receptors closely depends on the type of suicide—depressive or violent.

## Figures and Tables

**Table 1:** Summarized effects of 5-HT receptors on aggression and depression. Activating effect is shown by up arrow; suppressing effect is shown by down arrow; bidirectional effect is shown by double arrow; no effect is shown by straight line.

Receptor	Aggression	Depression
**5-HT_1A_**	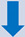	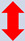
**5-HT_1B_**	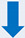	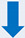
**5-HT_2A_**	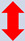	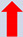
**5-HT_2B_**	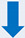	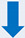
**5-HT_2C_**	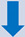	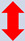
**5-HT_3_**	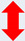	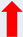
**5-HT_7_**	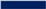	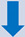

## Data Availability

Not applicable.

## Abbreviations

5-HT	Serotonin
SSRI	serotonin-selective reuptake inhibitor
MDD	Major Depressive Disorder
GPCR	G-protein coupled receptor
PET	positron emission tomography
siRNA	small interfering RNA
7-TMS	7-transmembrane-spanning receptors
cyclic GMP	cyclic guanosine monophosphate
BPD	borderline personality disorder
QTLs	Quantitative Trait Loci
TST	Tail Suspension Test
SNP	single nucleotide polymorphism
BD	bipolar affective disorder
ASC	antidepressant sensitive cataleptics
SS	Serotonin Syndrome
SERT	serotonin transporter
